# Fabrication of Curcumin@Ag Loaded Core/Shell Nanofiber Membrane and its Synergistic Antibacterial Properties

**DOI:** 10.3389/fchem.2022.870666

**Published:** 2022-03-16

**Authors:** Qiuxiang Wang, Songlin Liu, Wenjuan Lu, Pingping Zhang

**Affiliations:** School of Pharmacy & Pharmaceutical Sciences, Shandong First Medical University & Shandong Academy of Medical Sciences, Jinan, China

**Keywords:** photodynamic antibacterial, curcumin, silver nanoparticles, electrospinning, core/shell fibers, synergistic antibacterial properties

## Abstract

The core/shell structure nanofiber membrane loaded with curcumin and silver nanoparticles was prepared by coaxial electrospinning technology, which is a high-efficiency combined antibacterial material composed of photodynamic antibacterial agent and metal nanoparticle. As a photosensitizer, curcumin could generate singlet oxygen under laser irradiation. Silver nanoparticles have antibacterial properties, and could also enhance the singlet oxygen production of curcumin due to the metal-enhanced singlet oxygen effect, thereby producing a synergistic antibacterial effect. Compared with the antibacterial rate of uniaxial curcumin fiber membrane (45.65%) and uniaxial silver nanoparticle-loaded fiber membrane (66.96%), the antibacterial rate of curcumin@Ag core/shell structure fiber membrane against *Staphylococcus aureus* is as high as 93.04%. In addition, the antibacterial experiments show that the core/shell fiber membrane also has excellent antibacterial effects on *Escherichia coli*.

## Introduction

The overuse of antibiotics has led to acquired resistance of bacteria to most antibiotics, so there is an urgency to find new antibacterial methods or alternative strategies (Farhat et al., 2020; Yu et al., 2021; Zhao et al., 2021). There are mainly two approaches among the new antibacterial strategies. One is photodynamic antibacterial therapy (Wu et al., 2021), and the other is the use of metal nanoparticles (Godoy-Gallardo et al., 2021) or antimicrobial peptides (Qiu et al., 2021) to replace antibiotics. Photodynamic antimicrobial chemotherapy (PACT) kills bacteria through the combined action of light and photosensitizers (Liu et al., 2020; Sun et al., 2021). The mechanism of PACT is that the chromophore of the photosensitizer generates singlet oxygen and other reactive oxygen species (ROS) under light irradiation (Im et al., 2021; Xiao et al., 2021). ROS chemically attacks bacteria, and while bacteria at one site are naturally resistant to ROS attack, another site may be susceptible to the attack (Lyutakov et al., 2014).

The photosensitizer is the core and the key factor affecting the antibacterial effect of PACT (Wu et al., 2020). Natural photosensitizers with low toxicity and side effects are the hotspot of PACT research. Curcumin is a natural polyphenol found in plant rhizomes, which possesses a wide range of biological activities such as antioxidant (Hou et al., 2022), anti-inflammatory (Pontes-Quero et al., 2021), antitumor (Chen et al., 2021), antibacterial (Barros et al., 2021), antiviral (Dourado et al., 2021), and photosensitizing activities (Li et al., 2020). As a natural photosensitizer, curcumin can be activated by light at 400–500 nm to generate singlet oxygen to exert antibacterial effects (Hu et al., 2018). Feng et al. obtained a tough and biodegradable polyurethane-curcumin (PU-Cur) hydrogel with strongly antioxidant properties by *in-situ* copolymerization of Cur and PU. Under laser irradiation, the PU-Cur degradation solution can improve the inhibition rate of *Staphylococcus aureus*, which can promote wound healing (Feng et al., 2021). However, the singlet oxygen quantum yield of curcumin is relatively low, which makes it difficult to achieve the desired antibacterial effect. Combining curcumin with metal nanoparticles can improve the photosensitizer quantum yield by triggering the metal-enhanced singlet oxygen generation (MEO) effect (Mooi and Heyne, 2014). Among metal nanoparticles, silver nanoparticles (Ag NPs) are considered potential antibacterial agents because they can disrupt bacterial wall integrity (Fu et al., 2021; Wu et al., 2022), interfere with DNA replication (Li et al., 2011; Dhanalekshmi and Meena, 2016), and block adenosine triphosphate (ATP) synthesis in bacteria (Wang et al., 2018; Tong et al., 2020). Cao et al. used plant extract lignin as a reducing agent to synthesize Ag NPs (L-Ag NPs) with uniform size distribution by using one-step method, and found that L-Ag NPs have good antibacterial properties against gram-positive Candida albicans and gram-negative *Escherichia coli* (Cao et al., 2021). Ag NPs can enter the interior of bacteria to destroy the bacterial structure and exert antibacterial effects (Khalandi et al., 2017). The particle size and concentration of Ag NPs are the key factors affecting their antibacterial properties. Within a certain concentration range, the higher the concentration of Ag NPs, the better the antibacterial effect (Chen et al., 2019). However, too high concentration will cause Ag NPs to agglomerate and reduce their antibacterial properties (Zhang et al., 2022). Achieving uniform dispersion of Ag NPs at high concentrations is the premise to ensure their high-efficiency antibacterial properties.

Electrospinning is a technique of spraying a solution into a nanofiber membrane under a high-voltage electric field (Sun et al., 2019). Among them, uniaxial electrospinning, which is more commonly used, refers to electrospinning the drug solution through a single nozzle. He et al successfully prepared ciprofloxacin-loaded fibrous mats composed of different ratios of poly-ε-caprolactone (PCL) and polyethylene glycol (PEG) for wound healing by uniaxial electrospinning. The drug release could be controlled by changing the PEG ratio and the fiber mat geometry. The antibacterial effect of different fiber mats on gram-positive *Staphylococcus aureus* and gram-negative *Escherichia coli* was tested by the agar diffusion method. The experimental results showed that when the proportion of PEG was 10%, the fiber mat consisting of grids with a spacing of 0.8 mm had obvious inhibition zone (He et al., 2019). Pisani et al. loaded gentamycin sulfate (GS) into polylactic acid-co-polycaprolactone (PLA-PCL) electrospinning nanofibers for local drug delivery. Controlled drug release could reduce the side effects of GS and prolong the treatment effect. *In-vitro* antibacterial experiments showed that GS-loaded fiber mats had good antibacterial effects on both *Staphylococcus aureus* and *Escherichia coli* (Pisani et al., 2019). Wang et al. successfully prepared Cur-loaded zein fibers (zein-Cur) with encapsulation efficiency close to 100% using uniaxial electrospinning technology. When the content of Cur is 40% based on the weight of zein, the antibacterial rate of fiber against *Staphylococcus aureus* and *Escherichia coli* can reach 90%, and the antibacterial activity of zein-Cur fiber against *Staphylococcus aureus* is better than that of *Escherichia coli* (Wang et al., 2017). Uniaxial electrospinning is used for drug loading with the advantages of various types of loaded drugs or active ingredients and easy adjustment of the fiber structure (Li et al., 2021). When using uniaxial electrospinning to prepare electrospun antibacterial fibers co-loaded with multiple drugs or active ingredients, it is necessary to homogeneously mix multiple drug solutions or active ingredient solutions, which has relatively high requirements for electrospinning system. Coaxial electrospinning, one of the electrospinning technologies, is a technology in which two electrospinning solutions are put into different syringes and sprayed through a concentric needle device to obtain nanofibers with a core/shell structure (Rathore and Schiffman, 2021). The multi-component could be easily co-loaded by coaxial electrospinning. The active pharmaceutical ingredients that are unstable, require sustained release, and have poor spinnability can be used as the fiber core layer, while the shielding effect established by the core/shell structure can act to protect the active pharmaceutical ingredient (Luraghi et al., 2021).

Here, curcumin@Ag (Cur@Ag) core/shell nanofibers co-loaded with curcumin and Ag NPs were fabricated by coaxial electrospinning using polycaprolactone (PCL) and polyvinylpyrrolidone (PVP) solutions containing curcumin as the core layer electrospinning solution, and the PVP solution of Ag NPs as the shell layer electrospinning solution. The prepared nanofibrous membrane can achieve uniform loading of curcumin and Ag NPs. The photosensitizer curcumin generates singlet oxygen under 405 nm light irradiation, and Ag NPs improve the singlet oxygen quantum yield of curcumin through the metal-enhanced singlet oxygen generation effect, combined with the antibacterial effect of Ag NPs, thereby achieving a synergistic antibacterial effect. The as-prepared core/shell structured fibrous membrane has good antibacterial effect against *Staphylococcus aureus*, *Escherichia coli*, and methicillin-resistant *Staphylococcus aureus*.

## Experimental Section

### Materials

Silver nitrate, sodium chloride, and N, N-dimethylformamide (DMF) were purchased from Sinopharm Chemical Reagent Co., Ltd. Curcumin (Cur), polycaprolactone (PCL, Mw = 8×10^4^), and polyvinylpyrrolidone K-90 (PVP, Mw = 1.3×10^6^) were obtained from Dalian Meilun Biological Co., Ltd. Chloroform (CHCl_3_) was bought from Laiyang Kangde Chemical Co., Ltd., and ethanol (C_2_H_5_OH) was purchased from Tianjin Fuyu Fine Chemical Co., Ltd. Beef extract was obtained from Beijing Obosing Biotechnology Company. Tryptone was bought from OXOID, and agar powder was purchased from Solarbio. *Staphylococcus aureus* and *Escherichia coli* were obtained from Beijing Beina Chuanglian Institute of Biotechnology. Methicillin-resistant *Staphylococcus aureus* was purchased from Shanghai Biotechnology Center, and 2, 2, 6, 6-Tetramethylpiperidine (TEMP) was bought from Tongren Institute of Chemistry.

### Preparation of PCL/PVP@Cur Electrospun Fiber Membranes

Briefly, PCL/PVP (w/w, 0.6 g:0.4 g) was added in the mixture solvent of CHCl_3_/DMF (v/v, 8 ml:2 ml), and stirred at room temperature for 1.5 h until completely dissolved. Then, 0.15 g (15 wt% of the total polymer mass) curcumin was introduced and stirred at room temperature for 1 h in the dark to obtain a yellow homogeneous electrospinning solution C1. Similarly, 10 wt% of curcumin, 20 wt% of curcumin, and no curcumin were added to prepare electrospinning solutions C2, C3, and C4, according to the aforementioned method. Finally, the electrospinning solutions C1, C2, C3, and C4 were electrospun with a single nozzle by uniaxial electrospinning. The electrospinning process was performed at a constant flow rate of 0.2 mm/min under the conditions of 25°C temperature and 30% humidity by applying 20 kV voltage, and the nanofibers were collected on aluminum foil at a distance of 15 cm. The collected fiber membranes were dried at 50°C in the dark for 24 h to obtain electrospun fiber membranes CF1, CF2, and CF3 with single-loaded curcumin and a blank matrix fiber membrane CF4.

### Preparation of PVP@Ag Electrospun Fiber Membranes

First, 1.5 g of PVP was dissolved in the mixture solvent of C_2_H_5_OH/DMF (v/v, 7 ml:3 ml), and stirred at room temperature to reach uniformity. Then, 0.12 g (8 wt% of PVP mass) silver nitrate was added and stirred at room temperature in the dark to obtain a light yellow and transparent electrospinning solution S1. Similarly, 6 wt% and 10 wt% of silver nitrate were added to prepare electrospinning solutions S2 and S3, respectively, according to the aforementioned method. Finally, the electrospinning solutions S1, S2, and S3 were electrospun with a single nozzle. The electrospinning process was performed at a constant flow rate of 0.2 mm/min under the conditions of 25°C temperature and 30% humidity by applying 20 kV voltage, and the collect distance was 15 cm. The deposited fiber membranes were cross-linked at 150°C for 3 h, and then irradiated under a 254 nm UV lamp for 1 h to obtain electrospun fiber membranes SF1, SF2, and SF3 single-loaded with Ag NPs.

### Preparation of Cur@Ag Core/Shell Fiber Membranes

The electrospinning solutions C1 and S1 were used as core layer and shell layer electrospinning solutions, respectively, and coaxial electrospinning was conducted under the following electrospinning conditions: 20 kV positive pressure, 0.05 kV negative pressure, 25°C temperature, 30% humidity, the distance between the needle and the receiving plate was 15 cm, and the flow rate of electrospinning solution C1 and S1 was 0.2 mm/min and 0.4 mm/min, respectively. The deposited fiber membranes were cross-linked at 150°C for 3 h, and then irradiated under a 254 nm UV lamp for 1 h to obtain the electrospun fiber membrane CS co-loaded with Cur and Ag NPs.

### Characterization

The morphologies of the fiber membranes were characterized by field emission scanning electron microscopy (FE-SEM, JSM-6700F). The distribution of Ag NPs in fiber membranes was characterized by transmission electron microscopy (TEM, JEM-1011). The distribution of curcumin in fibrous membranes PCL/PVP@Cur was characterized by fluorescence microscopy (OLYMPUS DP80). The fiber diameter distribution of fiber membranes and the size distribution of Ag NPs in PVP@Ag fibrous membranes were analyzed by Image J software. The Fourier transform infrared (FTIR) analysis was performed with a Fourier transform infrared spectrometer (PerkinElmer).

Electron paramagnetic resonance (EPR) spectrometer (Bruker EMX PLUS) was used to measure the EPR signal intensity of 2, 2, 6, 6-tetramethylpiperidine oxide (TEMPO), which was formed after singlet oxygen generated by fiber membrane CF1 and CS was captured by 2, 2, 6, 6-tetramethylpiperidine (TEMP). The 3 × 3 cm fiber membrane was dissolved in 4 ml of pure water and sonicated for 5 min to obtain the sample solution, and then 100 μL of the sample solution was mixed with 200 μL of the singlet oxygen capture agent TEMP. After 10 min of darkness or light, the singlet oxygen was measured of the mixtures. Test conditions: center field 3,502.00 G, sweep width 100.0 G, power 6.325 mW, power attenuation 15.0 dB, frequency mon 9.829482 GHz, sweep time 30.00 s, modulation amplitude 1.000 G, and modulation frequency 100.00 kHz. The light source was a mercury lamp (500 W) with a 400 nm filter.

### Antibacterial Test

#### Antibacterial Experiment of Electrospun Fiber Membranes Against *Staphylococcus aureus*

In order to evaluate the antibacterial effect of PCL/PVP@Cur electrospun fiber membranes CF1, CF2, and CF3, the antibacterial study was carried out against gram-positive *Staphylococcus aureus* by using the colony counting method (Mahmud et al., 2020). First, a single colony of *Staphylococcus aureus* was inoculated into beef extract peptone liquid medium and placed in a shaker, which was cultured at 37°C and 220 rpm for 24 h to obtain the original bacterial solution. Then, the original bacterial solution was diluted to 1.0–3.0 × 10^7^ CFU/ml with sterile pure water to obtain a diluent solution. The CF1, CF2, and CF3 fiber membranes were cut into discs with a diameter of 1 cm and placed in eppendorf (EP) tubes containing 1 ml of diluent solution. Subsequently, the EP tubes were irradiated under a 405 nm UV lamp for 10 min and allowed to stand for 30 min to obtain the treatment solution. The EP tube containing diluent solution without fiber membrane was used as the blank group. 100 µl of the treatment solution was diluted 10^5^ times and spread evenly on a solid agar plate, and then incubated in the incubator at 37°C for 24 h. The number of viable colonies with samples was counted, and the antibacterial rates were calculated using the following formula:

$$\text{antibacterial}\;\text{rate}=\frac{N_{b}-N_{t}}{N_{b}}\times 100\%$$ (1)

where *N_b_* = number of colonies without sample, and *N_t_* = number of colonies with sample.

The PVP@Ag fiber membranes SF1, SF2, and SF3 were cut into discs with a diameter of 1 cm and placed in EP tubes containing 1 ml of diluent solution, and then allowed to stand for 40 min to obtain the treatment solution. The EP tube containing diluent solution without fiber membrane was used as the blank group.

The Cur@Ag core/shell fiber membrane CS was cut into a disc with a diameter of 1 cm and placed in an EP tube containing 1 ml of diluent solution. Subsequently, the EP tube was irradiated under a 405 nm UV lamp for 10 min and allowed to stand for 30 min to obtain the treatment solution. The EP tube containing diluent solution without fiber membrane was used as the blank group.

According to the antibacterial experimental method of PCL/PVP@Cur, the antibacterial rates of PVP@Ag fiber membrane and Cur@Ag core/shell fiber membrane were calculated to characterize their antibacterial properties.

#### Antibacterial Experiment of Electrospun Fiber Membranes CF1, SF1, and CS Against *Escherichia coli* and Methicillin-Resistant *Staphylococcus aureus* (MRSA)

The CF1, SF1, and CS fiber membranes were cut into discs with a diameter of 1 cm and placed in EP tubes containing 1 ml of *Escherichia coli* or MRSA dilutions, respectively. Then, the CF1 and CS fiber membranes were allowed to stand for 30 min after being irradiated under a 405 nm UV lamp for 10 min to obtain treatment solutions, and SF1 was left to stand for 40 min to obtain a treatment solution. The EP tube containing diluent solution without fiber membrane was used as the blank group. 100 µL of the treatment solutions drawn from the four treatment solutions were diluted 10^5^ times and spread evenly on solid agar plates, and then incubated in an incubator at 37°C for 24 h. The number of viable colonies with samples was counted, and the antibacterial rates of fiber membranes against *Escherichia coli* and MRSA, respectively, were calculated, according to Formula (1).

## Results and Discussion

### Characterization of PCL/PVP@Cur Fiber Membranes

**FIGURE 1:**
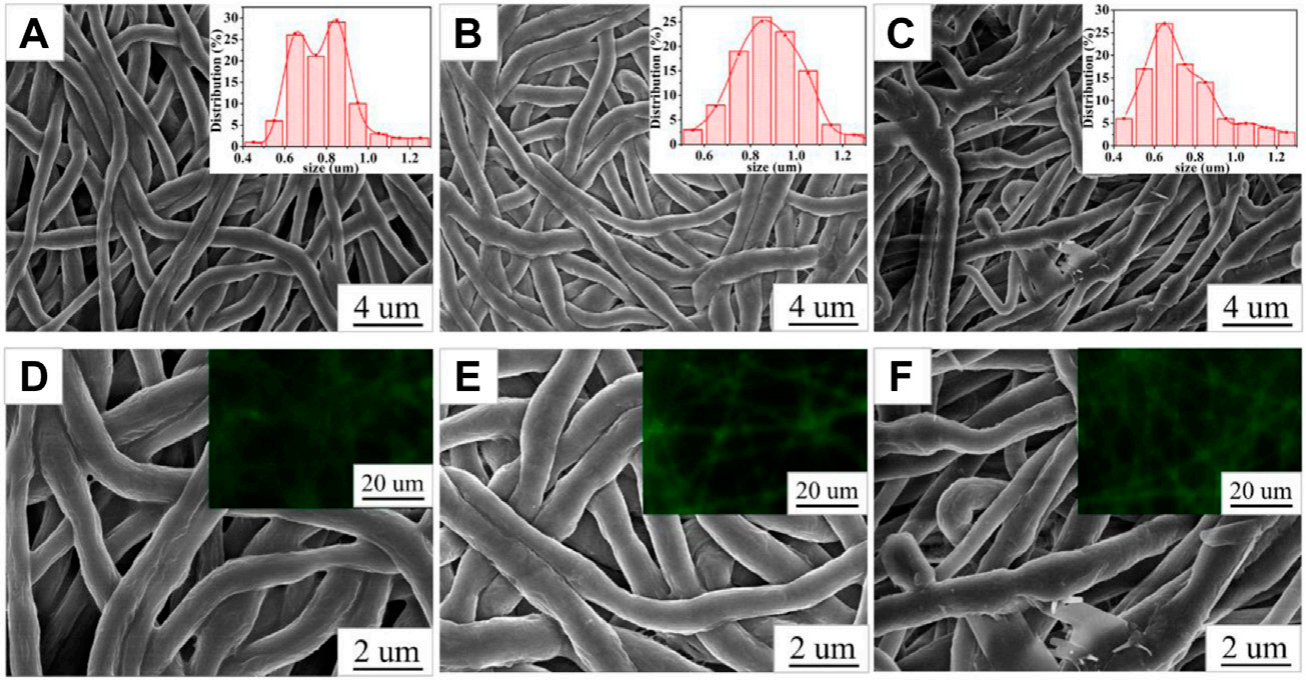
SEM images of CF2 **(A,D)**, CF1 **(B,E)**, and CF3 **(C,F)**; the insets in **(A,B, and C)** are the fiber diameter distribution graphs of CF2, CF1, and CF3; the insets in **(D,E, and F)** are the fluorescence micrographs of CF2, CF1, and CF3.

The SEM images of PCL/PVP@Cur electrospun fiber membranes are shown in Figure 1, and it can be seen that the prepared nanofibers were continuous. The surface of the fiber membranes CF2 and CF1 prepared at a curcumin concentration of 10% and 15%, respectively, was smooth without obvious defects (Figures 1A,B). Differently, the fiber membrane CF3 prepared when the concentration of curcumin was 20% had local fiber adhesion phenomenon, and the local fiber thickness difference was obvious (Figure 1C). The diameter distribution of the PCL/PVP@Cur electrospun fiber membranes was analyzed with Image J software. As shown in the insets of Figures 1A–C, the mean diameters of the fiber membranes CF2, CF1, and CF3 were 0.78 ± 0.15 um, 0.88 ± 0.15 um, and 0.74 ± 0.19 um, respectively. That is, when the curcumin concentration was 10% and 15%, the fiber diameter distribution was uniform and increased with the increase of curcumin concentration, whereas when the curcumin concentration increased to 20%, the fiber diameter distribution was uneven due to the existence of finer fibers, and the average diameter became smaller. Curcumin has fluorescent properties, so its distribution in the fibrous membrane can be observed by fluorescence microscopy. As shown in the insets of Figures 1D–F, a continuous strong fluorescence signal appeared throughout the fiber length, indicating that curcumin was uniformly distributed in the fibers (Tsekova et al., 2017).

**FIGURE 2:**
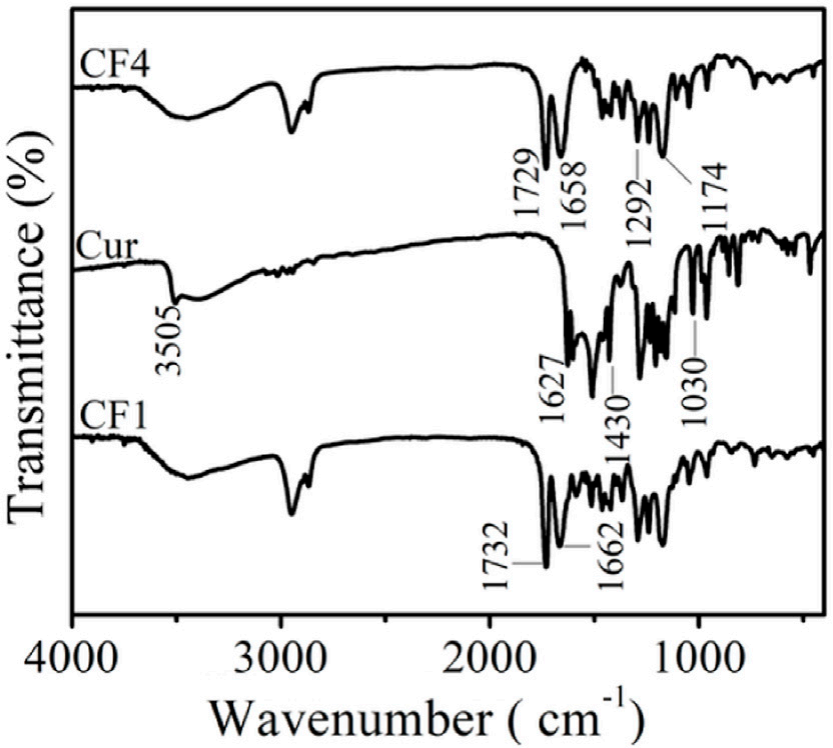
FTIR spectra of CF4, Cur, and CF1.

The infrared spectra of blank matrix fiber membranes CF4 and Cur and fiber membrane CF1 are shown in Figure 2. The absorption peak of curcumin at 3,505 cm^−1^ is attributed to the stretching vibration of phenolic hydroxyl O-H, and the peak at 1,627 cm^−1^ is due to the C=O stretching vibration. The peak at 1,430 cm^−1^ corresponds to the bending vibration of = C-H, and the absorption peak at 1,030 cm^−1^ is ascribed to the C-O-C stretching vibration of aromatic hydrocarbons (Liu et al., 2021). The peaks at 1729 cm^−1^ and 1,658 cm^−1^ in the CF4 fiber membrane correspond to the C=O stretching vibrations in the polymer PCL and PVP, respectively. The absorption peaks at 1,292 cm^−1^ and 1,174 cm^−1^ are assigned to the stretching vibration of C-N in the polymer PVP and the stretching vibration of C-O in polymer PCL, respectively. With the disappearance of the peak (3,505 cm^−1^) from the phenolic hydroxyl group of curcumin in the CF1 fiber membrane, the C=O stretching vibration peak in the PCL structure changed from 1729 cm^−1^ to 1732 cm^−1^, and the C=O stretching vibration in the PVP structure also changed from 1,658 cm^−1^ to 1,662 cm^−1^ (Yakub et al., 2020). It is speculated that the redshift of the peaks are caused by the formation of hydrogen bonds between the -OH in curcumin and the C=O structure in PCL and PVP, indicating that curcumin was successfully loaded into the fibers (Tsekova et al., 2017).

### Characterization of PVP@Ag Fiber Membranes

**FIGURE 3:**
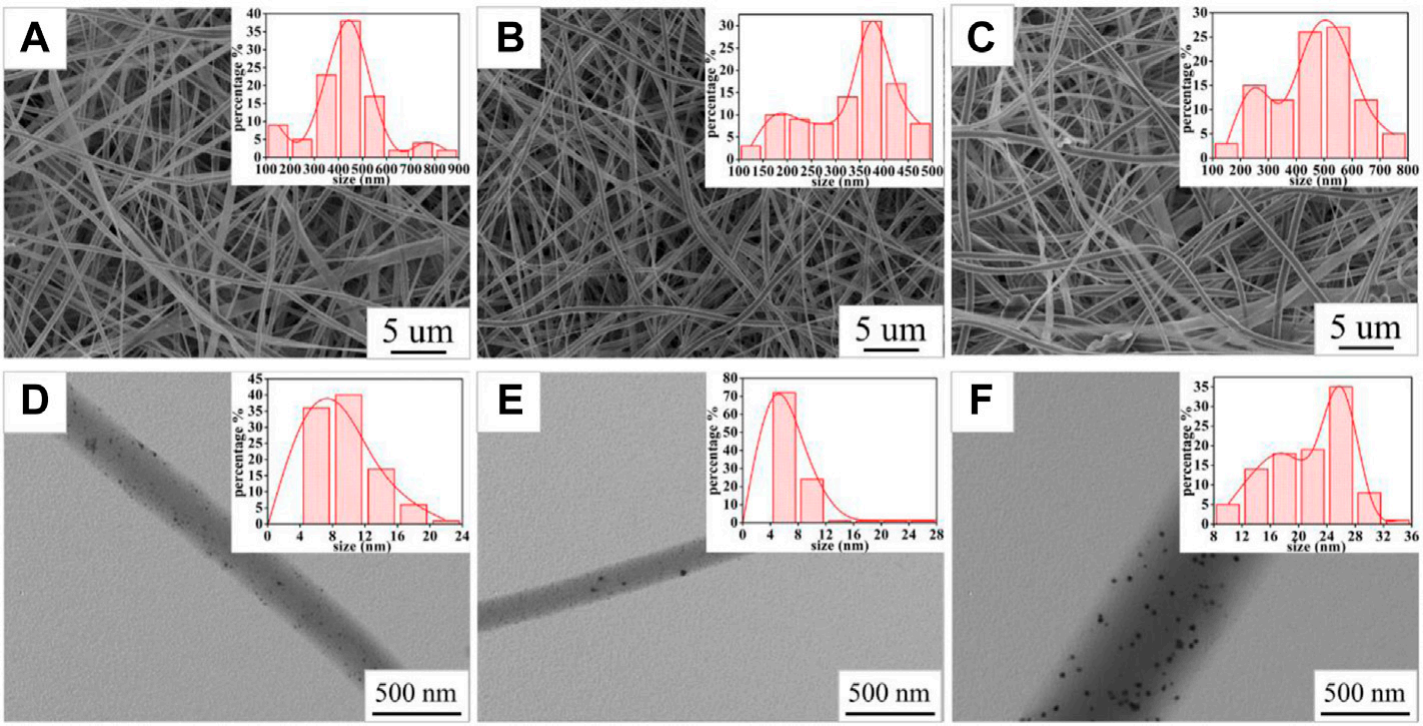
SEM images of SF2 **(A)**, SF1 **(B)**, and SF3 **(C)**; TEM images of SF2 **(D)**, SF1 **(E)**, and SF3 **(F)**; the insets in **(A,B,C)** are the fiber diameter distribution graphs of SF2, SF1, and SF3; the insets in **(D,E,F)** are the Ag NP size distribution graphs of SF2, SF1, and SF3.

The SEM images (Figures 3A–C) of the PVP@Ag electrospun fiber membranes showed that the prepared nanofibers were all continuous. The surface of the fiber membranes SF2 and SF1 prepared with the silver nitrate concentration of 6–8% was smooth and there was no slag ball (Figures 3A,B). Differently, the fiber membrane SF3 prepared when the silver nitrate concentration was 10% appeared as slag balls and ribbon fibers (Figure 3C). The TEM images (Figures 3D–F) of SF2, SF1, and SF3 indicated that Ag NPs obtained by silver nitrate reduction were dispersed in the fibers. Image J software was used to determine the fiber diameter distribution of the fiber membranes SF2, SF1, and SF3 and the size distribution of Ag NPs in the fibers. As shown in the insets of Figures 3A–C, the fiber diameters of the fiber membranes SF2, SF1, and SF3 were 428 ± 146 nm, 336 ± 93 nm, and 467 ± 141 nm, respectively. As shown in the insets of Figures 3D–F, the average sizes of Ag NPs in the fiber membranes SF2, SF1, and SF3 were 10.05 ± 3.48 nm, 7.98 ± 3.04 nm, and 21.75 ± 5.34 nm, respectively. The specific distribution of the size of Ag NPs is shown in Table 1. It can be seen from Table 1 that the proportion of Ag NPs of 4–8 nm in SF1 reaches 72%, and the smaller size was conducive to the antibacterial effect of Ag NPs (Wu et al., 2018; Calabrese et al., 2021). Based on the aforementioned analysis, when the silver nitrate concentration increased from 6% to 8%, the average fiber diameter and the average size of Ag NPs became smaller and more uniformly distributed, and the Ag NPs were evenly distributed in the fibers. However, when the silver nitrate concentration increased to 10%, the average fiber diameter and the average size of Ag NPs became larger and unevenly distributed, which may be caused by the aggregation of some Ag NPs into large particles.

**TABLE 1:** Size specific distribution of Ag NPs.

Fiber membrane	Ag NP size/nm	Ag NP size ratio (%)
SF1	4–8	72
	8–16	25
	16–28	3
SF2	4–8	36
	8–16	57
	16–24	7
SF3	8–16	19
	16–28	72
	28–36	9

### Characterization of Cur@Ag Core/Shell Fiber Membrane

**FIGURE 4:**
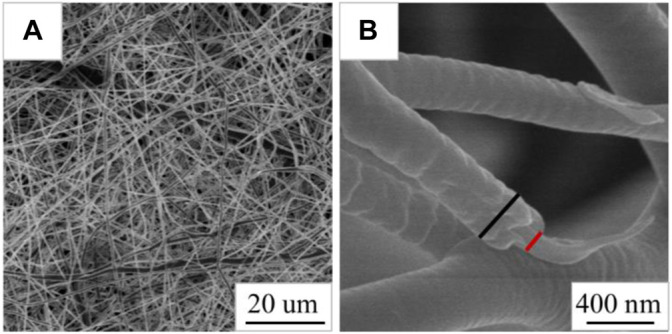
**(A)** SEM images of CS fibers, the marker in **(B)** is the core-shell structure of the fiber.

As shown in Figure 4A, the Cur@Ag fibers are continuous, and the fibers are randomly stacked and packed into a network structure. It can be clearly seen that the Cur@Ag fiber membrane has a core/shell structure from the mark in Figure 4B.

### Antibacterial Results of PCL/PVP@Cur Electrospun Fiber Membranes Against *Staphylococcus aureus*

**FIGURE 5:**
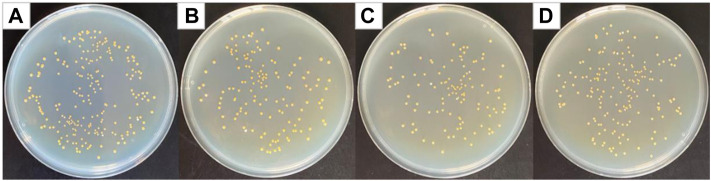
Photographs of antibacterial test result against *Staphylococcus aureus* **(A)** blank group, **(B)** CF2, **(C)** CF1, and **(D)** CF3.

Curcumin generates singlet oxygen under irradiation at 405 nm wavelength, which could cause significant structural damage to the membrane structure of *Staphylococcus aureus*, thereby increasing membrane permeability, leading to leakage of intra-bacterial substances and bacterial death (Yang et al., 2020). Figure 5 shows optical photographs of the antibacterial effect of PCL/PVP@Cur electrospun fiber membranes single-loaded with curcumin against *Staphylococcus aureus* for 24 h. It can be seen from the figure that the number of surviving colonies in the blank group and the group of fiber membranes CF2, CF1, and CF3 were 230, 140, 125, and 176, respectively. The calculation showed that the antibacterial rates of the CF2, CF1, and CF3 fiber membranes loaded with curcumin were 39.13, 45.65, and 23.48%, respectively. It can be seen from the antibacterial results that among the electrospinning fibers single-loaded with curcumin, the CF1 fiber membrane with a curcumin loading of 15% has the best antibacterial effect. Compared with CF2, CF1 has higher curcumin loading, so the antibacterial effect of CF1 is better than that of CF2. Among the PCL/PVP@Cur fiber membranes single-loaded with curcumin, CF3 has the highest curcumin loading, but its antibacterial performance is low, which may be related to the adhesion of its fibers and the obvious difference in local fiber diameters (Figure 1C).

### Antibacterial Results of PVP@Ag Electrospun Fiber Membranes Against *Staphylococcus aureus*

**FIGURE 6:**
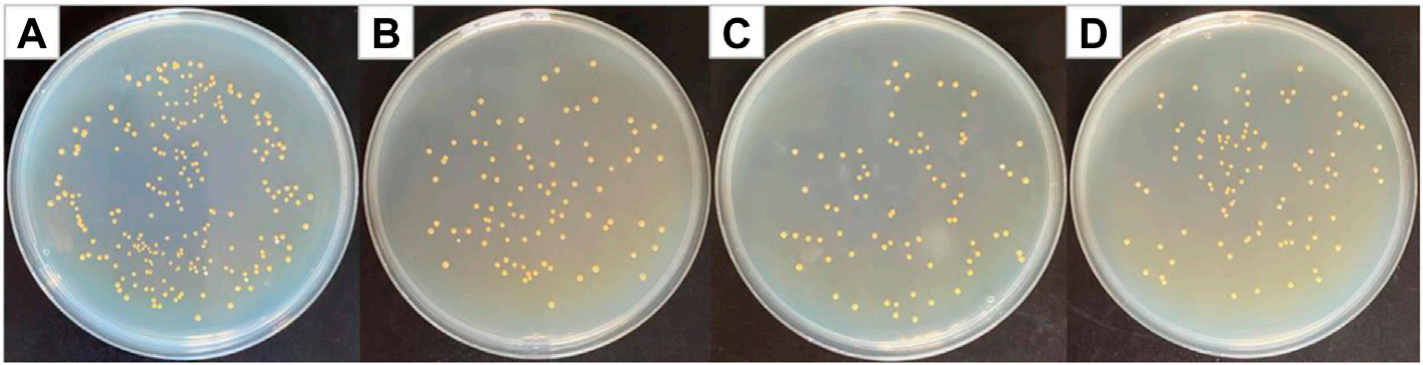
Photographs of antibacterial test result against *Staphylococcus aureus* **(A)** blank group, **(B)** SF2, **(C)** SF1, and **(D)** SF3.

Figure 6 shows optical photographs of the antibacterial effect of PVP@Ag electrospun fiber membranes single-loaded with Ag NPs against *Staphylococcus aureus*. As shown in Figure 6, the number of surviving colonies in the blank group and the group of fiber membranes SF2, SF1, and SF3 were 230, 87, 76, and 97, respectively. The calculation showed that the antibacterial rates of the SF2, SF1, and SF3 fiber membranes single-loaded with Ag NPs were 62.17, 66.96, and 57.83%, respectively. The results exhibited that when the amount of silver nitrate increased from 6 to 8%, the antibacterial effect of the obtained SF1 fiber membrane was better than that of SF2, but the antibacterial effect of the obtained SF3 fiber membrane decreased when the concentration increased to 10%. It can be seen from Figure 3 and Table 1 that the Ag NPs obtained after the reduction of silver nitrate in the SF1 fiber membrane are small in size and concentrated in 4–16 nm, which accounts for 97%, and is uniformly dispersed in the fiber. Small-sized monodispersed Ag NPs attribute to the superior antibacterial properties of SF1 (Marambio-Jones and Hoek, 2010; Zhou et al., 2022).

### Antibacterial Results of Cur@Ag Core/Shell Fibrous Membrane Against *Staphylococcus aureus*, *Escherichia coli*, and MRSA

**FIGURE 7:**
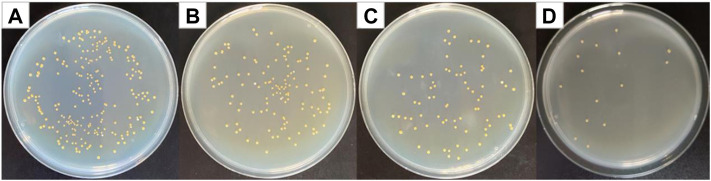
Photographs of antibacterial test result against *Staphylococcus aureus* **(A)** blank group, **(B)** CF1, **(C)** SF1, and **(D)** CS.

Figure 7 shows optical photographs of the antibacterial effect of Cur@Ag core/shell fiber membrane against *Staphylococcus aureus*. As shown in Figure 7, the number of surviving colonies in the Cur@Ag core/shell fiber membrane (CS) was 16, and its bacteriostatic rate was as high as 93.04%. Compared with the antibacterial rate 45.65% of the fiber membrane CF1 single-loaded with curcumin and the antibacterial rate 66.96% of the fiber membrane SF1 single-loaded with Ag NPs, the antibacterial rate of Cur@Ag core/shell fiber membrane CS was significantly increased, indicating that curcumin and Ag NPs in the Cur@Ag core/shell fibrous membrane exhibited a clear synergistic inhibitory effect on *Staphylococcus aureus*.

**FIGURE 8:**
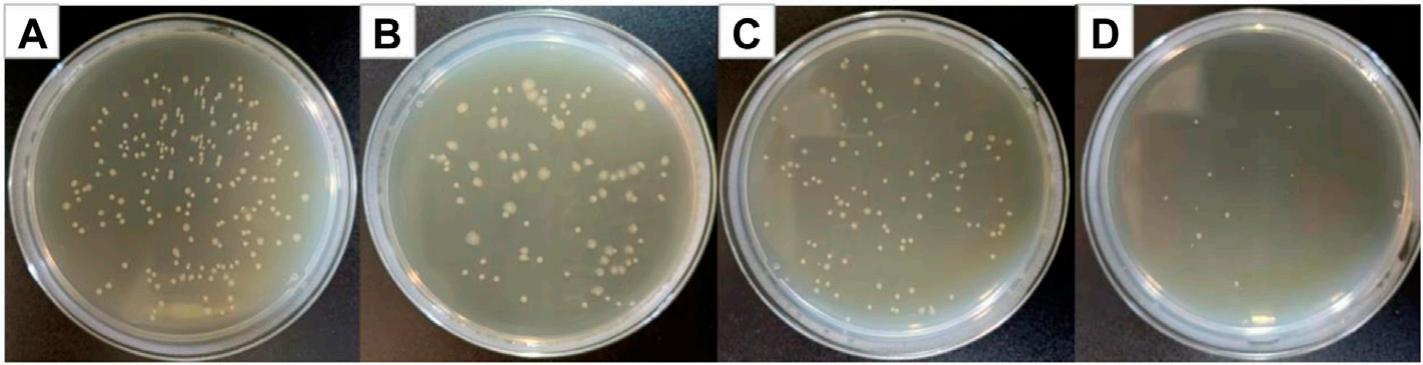
Photographs of antibacterial test result against *Escherichia coli* **(A)** blank group, **(B)** CF1, **(C)** SF1, and **(D)** CS.

Figure 8 shows optical photographs of the antibacterial effect of Cur@Ag core/shell fiber membrane against *Escherichia coli*. As shown in Figure 8, the number of surviving colonies in the blank group and the group of fiber membranes CF1, SF1, and CS were 209, 81, 101, and 15, respectively. The calculation showed that the antibacterial rates of fiber membrane CF1, SF1, and CS were 61.24%, 51.67%, and 92.82%, respectively. Compared with the antibacterial rate 61.24% of the fiber membrane CF1 single-loaded with curcumin, and the antibacterial rate 51.67% of the fiber membrane SF1 single-loaded with Ag NPs, the antibacterial rate of Cur@Ag core/shell fiber membrane CS was significantly increased, indicating that curcumin and Ag NPs could play an obvious synergistic inhibitory effect on *Escherichia coli*.

**FIGURE 9:**
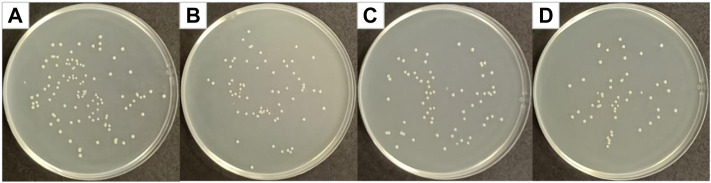
Photographs of antibacterial test result against MRSA **(A)** blank group, **(B)** CF1, **(C)** SF1, and **(D)** CS.

Figure 9 shows optical photographs of the antibacterial effect of Cur@Ag core/shell fiber membrane against MRSA. As shown in Figure 9, the number of surviving colonies in the blank group and the group of fiber membranes CF1, SF1, and CS were 108, 68, 66, and 51, respectively. The calculation showed that the antibacterial rates of fiber membrane CF1, SF1, and CS were 37.04, 38.89, and 52.78%, respectively. Compared with the antibacterial rate 37.04% of the fiber membrane CF1 single-loaded with curcumin, and the antibacterial rate 38.89% of the fiber membrane SF1 single-loaded with Ag NPs, the antibacterial rate of Cur@Ag core/shell fiber membrane CS was increased, indicating that curcumin and Ag NPs could also play a synergistic inhibitory effect on MRSA.

**FIGURE 10:**
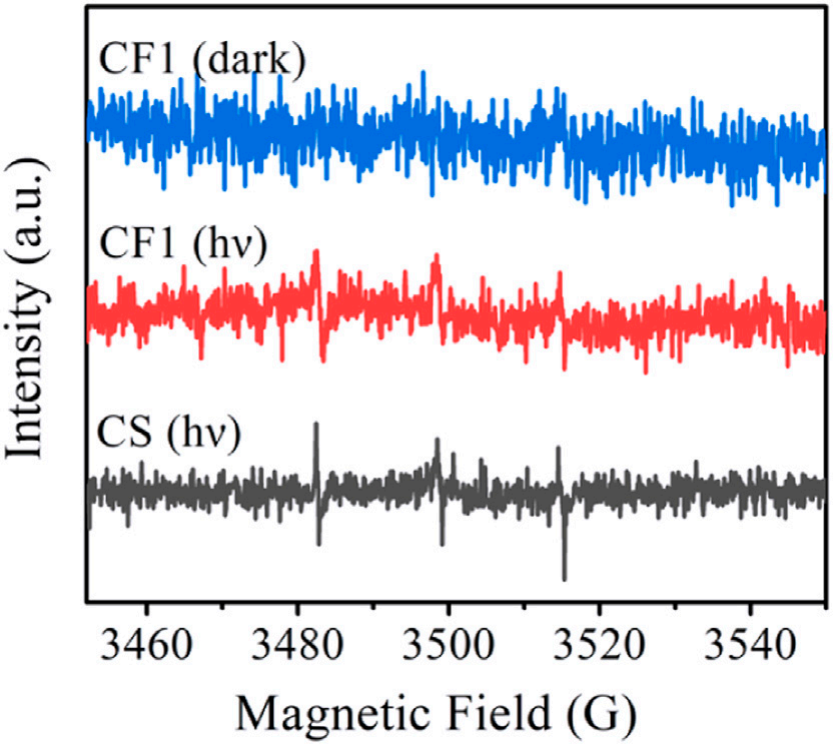
EPR spectra of ^1^O_2_ captured by TEMP after CF1 was dispersed in water under dark and light for 10 min, and CS was dispersed in water under light for 10 min.

The singlet oxygen test was performed on the fiber membranes CF1 and CS to explore the synergistic antibacterial mechanism of curcumin and Ag NPs. The test results are shown in Figure 10.

The singlet oxygen induced by the dispersion of fiber membrane CF1 and fiber membrane CS in pure water can be captured by TEMP and form stable 2, 2, 6, 6-tetramethylpiperidine oxide (TEMPO). The ability of curcumin to generate singlet oxygen in the fiber membrane under different conditions can be known by analyzing the electron paramagnetic resonance (EPR) signal intensity of TEMPO. As shown in Figure 10, for CF1, no EPR signal of TEMPO was detected under dark conditions, while three weak EPR signals of TEMPO were observed under light conditions, indicating that curcumin could be induced to produce singlet oxygen under light conditions. Under light conditions, CS detected the strong EPR signals, revealing that the presence of Ag NPs can promote the generation of curcumin singlet oxygen. Ag NPs exerted the metal-enhanced singlet oxygen production effect and improved the singlet oxygen yield (Yu et al., 2020; Wang et al., 2021), so that the antibacterial effect of fiber membrane CS was better than that of CF1 and SF1, and the combined inhibitory effect of Cur and Ag NPs on *Staphylococcus aureus*, *Escherichia coli*, and MRSA was achieved.

## Conclusion

In this study, the curcumin@Ag loaded core/shell nanofiber membrane was constructed by coaxial electrospinning technology. Cur and Ag NPs were uniformly distributed in the core and shell layers of the fiber membrane, respectively. Ag NPs improve the singlet oxygen yield of curcumin through the metal-enhanced singlet oxygen generation effect. The antibacterial experiments showed that compared with the fiber membranes single-loaded with curcumin and Ag NPs, curcumin@Ag loaded core/shell nanofiber membrane exhibited excellent antibacterial effects on both *Staphylococcus aureus* and *Escherichia coli*, and the antibacterial rates reached 93.04% and 92.82%, respectively. At the same time, curcumin@Ag loaded core/shell nanofiber membrane also has synergistic antibacterial effect on methicillin-resistant *Staphylococcus aureus*. In the future, the elucidation of the potential antibacterial mechanism by examining the morphological changes and DNA damage of bacterial cells, and the enhancement of antibacterial effect against drug-resistant bacteria would further favor the application of prepared core/shell nanofiber antimicrobial materials in the field of synergistic antibacterials.

## Data Availability

The original contributions presented in the study are included in the article/Supplementary Material, further inquiries can be directed to the corresponding author.
